# Selections

**Published:** 1896-10

**Authors:** 


### Selections.

### FATAL ACUTE POISONING BY COCAINE.

Dr. O. H. Garland, of Leith, relates the following case, of some medico-legal interest owing to the few fatal cases of the kind recorded: "About 3.30 p.m., on Monday, October 7, 1895, at the request of the authorities, I made a superficial examination of the body of a young woman who had at about seven a.m. swallowed, for the relief of toothache, upward of two fluidrachms (eight cubic centimetres) of a ten per-cent. solution of the hydrochlorate of cocaine,—the equivalent of twelve to fifteen grains (0.78 to 1 gramme) of the alkaloid. She had almost immediately afterwards suffered from vertigo, followed by epileptiform convulsions, nine in number, occurring in somewhat quick succession, and died within forty minutes. She appeared to be about seventeen years of age, and the body was well nourished. The trunk still contained a considerable degree of warmth, but the extremities were cold, *rigor mortis* being well developed in all of them. The face, lips, and anterior surface of the body were very pallid and wax-like, while on the dependent portions well-marked cutaneous hypostasis was present. The expression of countenance was placid. The pupils were dilated, and the conjunctivæ were not injected. The teeth were firmly clinched. On the following day (Tuesday), as further instructed, I made a post-mortem examination. The external appearances remained as described. Frothy mucus, slightly blood-stained, was freely exuding from the right nostril. The brain was anæmic, but otherwise normal. The meninges were deeply congested. The heart was healthy in substance, and all the valves were found to be competent. The right ventricle contained a small quantity of dark-colored fluid blood; the left ventricle was quite empty. Both ventricular walls were relaxed. The lungs were too much congested throughout, and everywhere highly crepitant. A considerable amount of frothy mucus, in part blood-stained, was present in the bronchi. The liver, spleen, and kidneys were hyperæmic, but otherwise normal. The stomach and contents were removed, and preserved intact for expert analysis; consequently there was no opportunity afforded for inspection of the mucous surface of that organ. The bladder contained from three to four ounces of urine. All the other organs were normal." The case, according to the author, lends support to the view of Mannheim, that fifteen grains (one gramme) of cocaine constituted a fatal dose. In the present state of our knowledge it is, however, practically impossible to state the smallest lethal dose, seeing that a dose of two-thirds grain (0.04 gramme) has caused death, and so minute a dose as 0.01 grain (0.00065 gramme) has given rise to symptoms threatening life.—*Lancet.*

---

### FORMULA FOR LOCAL ANÆSTHESIA.

A MIXTURE of chloroform ten parts, ether fifteen parts, and menthol one part, used as a spray, is recommended as an excellent and prompt means for obtaining local anæsthesia, lasting for about five minutes.—*Boston Medical and Surgical Journal.*

---

### THE MEDICAL PROFESSION IN THE UNITED STATES.

ACCORDING to the Bulletin on "occupations" of the eleventh census just issued, there were in the United States, in the year 1890, 104,803 physicians and surgeons, or about one to every 600 of the population. The total number of wage-earners of all occupations is placed at 22,735,661. There were 89,630 lawyers, 88,295 clergymen, 59,090 nurses and midwives, 17,498 dentists, and 9900 undertakers.—*Medical News.*

